# Comparison of Intranasal Outer Membrane Vesicles with Cholera Toxin and Injected MF59C.1 as Adjuvants for Malaria Transmission Blocking Antigens AnAPN1 and Pfs48/45

**DOI:** 10.1155/2016/3576028

**Published:** 2016-04-27

**Authors:** Michael Pritsch, Najib Ben-Khaled, Michael Chaloupka, Sebastian Kobold, Nicole Berens-Riha, Annabell Peter, Gabriele Liegl, Sören Schubert, Michael Hoelscher, Thomas Löscher, Andreas Wieser

**Affiliations:** ^1^Division of Infectious Diseases and Tropical Medicine, Medical Center of the University of Munich (LMU), 80802 Munich, Germany; ^2^Department of Bacteriology, Max von Pettenkofer Institute (LMU), 81337 Munich, Germany; ^3^German Center for Infection Research (DZIF), Partner Site Munich, 80802 Munich, Germany; ^4^Center of Integrated Protein Science Munich (CIPS-M) and Division of Clinical Pharmacology, Department of Internal Medicine IV, University of Munich (LMU), 80337 Munich, Germany; ^5^German Center for Lung Research, Munich, Germany; ^6^College of Public Health and Medical Science, Jimma University, Jimma, Ethiopia

## Abstract

Purified protein vaccines often require adjuvants for efficient stimulation of immune responses. There is no licensed mucosal adjuvant on the market to adequately boost the immune response to purified antigens for intranasal applications in humans. Bacterial outer membrane vesicles (OMV) are attractive candidates potentially combining antigenic and adjuvant properties in one substance. To more precisely characterize the potential of* Escherichia coli* OMV for intranasal vaccination with heterologous antigens, immune responses for AnAPN1 and Pfs48/45 as well as ovalbumin as a reference antigen were assessed in mice. The intranasal adjuvant cholera toxin (CT) and parenteral adjuvant MF59C.1 were used in comparison. Vaccinations were administered intranasally or subcutaneously. Antibodies (total IgG and IgM as well as subclasses IgG1, IgG2a, IgG2b, and IgG3) were measured by ELISA. T cell responses (cytotoxic T cells, Th1, Th17, and regulatory T cells) were determined by flow cytometry. When OMV were used as adjuvant for intranasal immunization, antibody and cellular responses against all three antigens could be induced, comparable to cholera toxin and MF59C.1. Antigen-specific IgG titres above 1 : 10^5^ could be detected in all groups. This study provides the rationale for further development of OMV as a vaccination strategy in malaria and other diseases.

## 1. Introduction

Vaccines are one of the most cost-effective measures in the field of public health and greatly reduce disease, disability, death, and inequity worldwide [1, 2]. Their pivotal role has been demonstrated in infectious disease elimination campaigns (e.g., against smallpox, polio, or measles). However, due to the diversity of pathogens and their specific requirements for immune elimination or prevention, vaccination strategies cannot be readily translated from one disease to another, but they rely on a fine definition of protection and a good understanding of the immunological mechanisms underlying each vaccination [3].

Most vaccine formulations require adjuvant substances to boost immune responses, which have to be chosen according to their ability to induce the desired type of immune response without causing disproportional toxicity [4]. Another important consideration for vaccines is their application route. The most commonly used parenteral injection depends on the presence of sterile needles and medical personnel, which are major issues in parts of the world where infectious diseases are most prevalent. Mucosal vaccination strategies have the potential to overcome these limitations and are thereby in focus of vaccine research [5]. However, not every potential application route for mucosal vaccination may be socially and culturally acceptable. Oral or respiratory mucosal immunization strategies may have the highest acceptance and may circumvent many of the shortcomings of parenteral injections. Nonreplicating particles or recombinant proteins in combination with mucosal adjuvants can evoke mucosal and systemic immune responses [5]. Immune responses to vaccines also differ significantly in their ability to induce reactive T cells. Mucosal—especially intranasal (i.n.)—vaccines have been shown to produce strong T cellular responses [5]. These exquisite properties of mucosal vaccines are in sharp contrast to their current use, as only few vaccines have been approved so far for this indication (e.g., against polio, typhoid fever, or uropathogenic* E. coli* delivered orally or flu delivered as nasal spray); all of these consist of attenuated or inactivated pathogens. A major reason for this discrepancy is the lack of proper adjuvant substances [6].

Bacterial outer membrane vesicles (OMV) are particles about 100 nanometres in diameter produced by gram-negative organisms during growth. OMV encompass gram-negative outer membrane including transmembrane proteins as well as periplasmic matter. OMV are inherently potent immune-stimulators and are able to penetrate mucosal membranes as potent danger signal for innate immunity. Together with heterologous antigens, they can be used as adjuvant substances to promote immunity to these antigens [7–9]. Several studies have investigated the properties of* Neisseria* spp. OMV as vaccines as well as adjuvants [10–12]. It is currently unresolved how OMV would compare with conventional adjuvants in their capacity to induce immune reactions against malaria vaccine candidates.

Malaria has a huge impact on public health worldwide, causing 700,000 [13] to 1.2 million [14] deaths annually. Thus, there is a need to develop efficient strategies, such as transmission blocking vaccines. Among several others, two malaria antigens are deemed suitable for transmission blockade: (i) the* Anopheles* alanyl aminopeptidase N (AnAPN1) of the midgut of* Anopheles* mosquitoes has been found to play a critical role in* Plasmodium falciparum* and* Plasmodium vivax* ookinete invasion. Immunization against AnAPN1 was shown to inhibit* Plasmodium* replication in mosquitoes and with this stop transmission [15–18]; (ii) in the malaria parasite sexual stage, prefertilization gametocyte antigen Pfs48/45 was described to play a critical role in male gamete fertility, and vaccination can induce potent malaria transmission blocking antibodies in mice and nonhuman primates [19–27].

To characterize* E. coli* OMV as mucosal vaccine adjuvant in comparison to established adjuvants, mice were vaccinated with either AnAPN1, Pfs48/45, or ovalbumin (OVA) using intranasal OMV, intranasal cholera toxin (CT), or subcutaneous MF59C.1, respectively. Humoral and cellular responses were measured after 31 days. This study demonstrates that OMV elicit robust humoral and cellular immune responses against the tested antigens. Antibody titres were found to be comparable between the vaccination groups using the three different adjuvants. This study provides evidence for further evaluation of OMV for vaccination against malaria and other infectious diseases.

## 2. Materials and Methods

### 2.1. Construction, Expression, and Purification of Proteins

The amino acid sequences of AnAPN1 and Pfs48/45 were used as target antigens for the study. In case of AnAPN1, a 135-amino-acid fragment containing the residues 61–194 located downstream of the N-terminus of mature AnAPN1 was used as to preserve also the C-terminal transmission blocking peptide 9, as described previously [15, 17]. In case of Pfs48/45, the native sequence (accession number AF356146) lacking the N-terminal signal sequence (amino acid residues 1–27) and the C-terminal anchor (amino acid residues 428–448) was used as described before [26]. Amino acid sequences were reversely translated into coding DNA sequences taking into account the optimized codon usage for* Enterobacteriaceae* as well as RNA secondary structures and other prominent sequences such as chi sites (DNAStar Inc., Madison, WI, USA). The resulting DNA sequences were synthetically generated by GeneArt (Regensburg, Germany). Synthetic genes were produced including flanking restriction sites for simplified cloning. For purification, the well-characterized 6-His tag was fused to the N-terminus of each vaccine protein. The gene constructs were subcloned into pASK-iba37+ Plasmids (IBA-Lifesciences, Goettingen, Germany), using the restriction sites KpnI/PstI for AnAPN1 and KpnI/HindIII for Pfs48/45, respectively. Final plasmids were electroporated into the* E. coli* strain Top-10 and induced with anhydrotetracycline (AHT). Induction was performed as described by the manufacturer of the plasmid (IBA-Lifesciences, Goettingen, Germany). In brief, the specific strain was grown in LB containing ampicillin at a final concentration of 100 *μ*g/mL at 37°C under agitation. After an OD_600_ of 0.5 was reached, AHT was added to a final concentration of 0.2 *μ*g/mL. The induction was performed for 3 h at 37°C under agitation. Bacterial cell pellets were harvested by centrifugation of the induced culture at 5000 ×g at 4°C for 20 min and subsequently lysed in lysis buffer. The proteins AnAPN1 and Pfs48/45, which were tagged with a 6-His tag at the N-terminal side, were purified using Ni-nitrilotriacetic acid (Ni-NTA) columns. After several wash steps, the proteins were gel filtered to ensure high purity. Elution was performed under denaturing conditions with buffer containing 5 mM Imidazole and 7 M urea.

Cell culture grade, pyrogen-free OVA was purchased from Sigma-Aldrich (St. Louis, MO, USA); control protein His-DHFR-m45 stock solution was obtained from previous productions [28].

### 2.2. SDS-PAGE and Immuno-Blot Analysis

Discontinuous one-dimensional sodium dodecyl sulfate-polyacrylamide gel electrophoresis (SDS-PAGE) was performed with a Protran II Mini-Vertical unit (Bio-Rad, Munich, Germany). After electrophoresis, gels were either stained with Coomassie Blue or transferred to a nitrocellulose membrane (Trans Blot Cell; Bio-Rad, Munich, Germany). The blot was blocked with 3% (w/v) bovine serum albumin (BSA) in phosphate buffered saline (PBS) supplemented with 0.5% (v/v) Tween and incubated with diluted mouse serum. Preimmune or postimmune sera were used, respectively, followed by goat anti-mouse immunoglobulin (A, M, or G) peroxidase conjugate (Sigma-Aldrich, Germany). Blots were developed using enhanced chemiluminescence (ECL) detection reagents (Amersham Pharmacia Biotech/GE, Freiburg, Germany). Serum dilutions for IgM were 1 : 1,000 and for IgG 1 : 20,000. Dilutions of secondary antibodies used for blot development were 1 : 4,000 for IgG and 1 : 2,000 for IgM.

### 2.3. Production of Adjuvants and Quantification of OMV

Cholera toxin (CT) was obtained from Quadratech Diagnostics Ltd. (Epsom, UK). MF59C.1 was produced by Marien-Apotheke München (Munich, Germany). OMVs were obtained by harvesting from the supernatant of growing bacterial cultures, as described previously [29]. In brief,* E. coli* strain AW OMV01 (Wieser at al., manuscript in preparation) bacteria were inoculated into prefiltered LB broth (0.22 *μ*m pore size filters; Millipore/Merck, Darmstadt, Germany) and grown under vigorous agitation and aeration at 37°C to an OD_600_ up to 2.0. Bacteria were removed from the culture by centrifugation. The supernatant was sterile filtered with 0.45 *μ*m pore size filter (Millipore/Merck, Darmstadt, Germany). The OMVs were enriched from filtrates by ultracentrifugation at 150,000 ×g at 4°C for 3 h. The resulting translucent pellet was suspended in sterile phosphate buffered saline (PBS) and stored at 4°C in the fridge until use for a maximum of 3 days. Quantification of OMV was performed as described before [29] using flow cytometry as well as Bradford protein measurements.

### 2.4. Vaccination of Animals

Animal experiments were carried out in strict accordance with the recommendations in the guidelines of the Federation of European Laboratory Animal Science Associations. On June 1st, 2012, the accountable German authority (Government of Upper Bavaria, Munich, Germany) approved the study protocol. Animals used for vaccinations were 6- to 8-week-old female pathogen-free BALB/c mice (Janvier, Saint-Berthevin, France), 14.9 g to 20.3 g of weight. All animals were housed under specific pathogen-free (SPF) conditions in individually ventilated positive pressure cabinets (Tecniplast, Hohenpeissenberg, Germany) with controlled temperature and humidity as well as strict 12 h day/night cycle. Bedding was autoclaved before use; sterile water and sniff-extrudated food were offered* ad libitum*. Each experiment was repeated at least twice with 5 mice in each group. I.n. vaccination was performed on days 0, 3, 5, and 21. For vaccination, mice were anesthetized with isoflurane 4% for a few seconds in the anaesthesiology chamber of a narcosis device (XGI-8 Gas Anesthesia System; Xenogen Corporation, Alameda, Ca, USA). Anaesthetized mice were handled inside the laminar flow hood and the vaccine was delivered into each nostril using 20 *μ*L Eppendorf GE-Loader tips (Eppendorf, Hamburg, Germany). To increase stiffness, they were trimmed with sterile scissors to a total length of about 30 mm before use. Each nostril of the mouse was individually probed and 5 *μ*L of the aqueous vaccination solution was administered. The vaccine contained either (i) 10 *μ*g of the respective vaccine protein (AnAPN1, Pfs48/45, or OVA) plus CT (Quadratech, Surrey, United Kingdom) (0.5 *μ*g/10 *μ*L) or (ii) 10 *μ*g of the respective vaccine protein (AnAPN1, Pfs48/45, or ovalbumin) plus 5 *μ*L OMVs or (iii) CT (0.5 *μ*g/10 *μ*L) or (iv) 5 *μ*L OMVs. Each (i–iv) solution was prepared in PBS with adjusted pH of 7.4. All solutions were freshly prepared and mixed again directly prior to the application. As an application control PBS was used (v).

For parenteral application, the subcutaneous (s.c.) vaccination route was chosen inspired by the data of Valensi et al. [30]. In pilot experiments, we could detect robust immune responses using subcutaneous immunization rather than intramuscular while maintaining better tolerability on the side of the laboratory animal. Doses were administered on days 0, 7, and 21; a total amount of 20 *μ*L of an aqueous solution was inoculated into the scruff of the neck, containing either (i) 10 *μ*g of the respective vaccine protein (AnAPN1, Pfs48/45, or OVA) plus 10 *μ*g MF59C.1 or (ii) 10 *μ*g MF59C.1, each (i, ii) diluted in PBS, or (iii) PBS only as application control.

All mice were sacrificed on day 31 to obtain mouse sera and splenocytes to investigate the evoked immune responses. Throughout the experiments, mice were monitored daily for abnormalities of behaviour. The weight was determined on a weekly basis.

### 2.5. Measurement of the Immune Responses in the Mouse

The antibody response as well as the T cellular response to the vaccinations was analysed with different methods.

**Figure 1:**
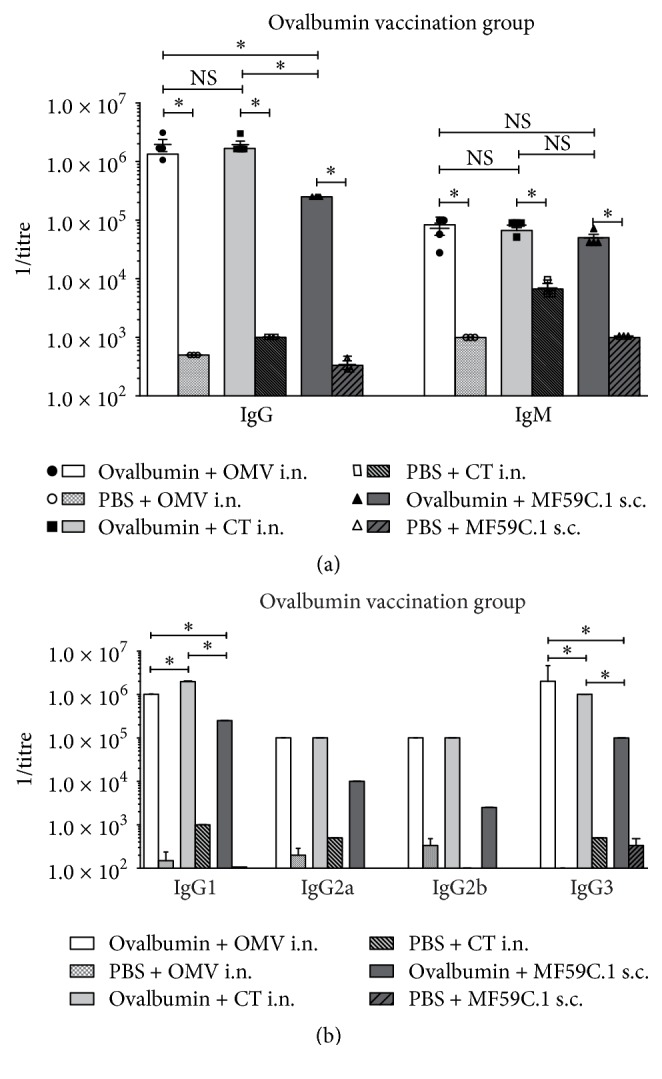
Characterization of the humoral response to ovalbumin vaccination. Each column depicts a representative vaccination group consisting of five mice, respectively. (a) depicts whole IgG and IgM titres in direct comparison between the three adjuvants. Single mouse as well as mixed mouse serum data is shown including standard deviations. For control groups, only three individual mouse sera were tested additionally to the mixed serum. (b) shows IgG1, -2a, -2b, and -3 titres of mixed mouse sera in comparison for the three adjuvant substances including controls. Titres of <10^2^ have not been determined and thus are not depicted. NS = nonsignificant; *∗* = significant difference *p* < 0.05; OMVs = bacterial outer membrane vesicles; CT = cholera toxin; i.n. = intranasal vaccination; s.c. = subcutaneous vaccination.

**Figure 2:**
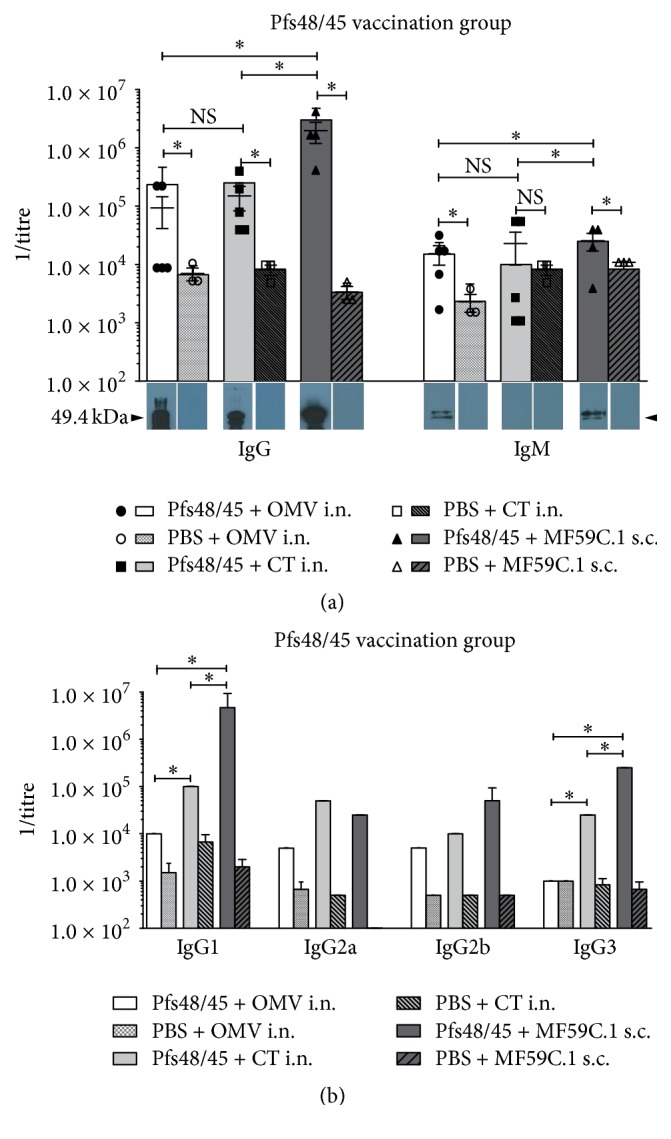
Characterization of the humoral response to Pfs48/45 vaccination. Each column depicts a representative vaccination group consisting of five mice, respectively. (a) depicts whole IgG and IgM titres in direct comparison between the three adjuvants. Single mouse as well as mixed mouse serum data is shown including standard deviations. For control groups, only three individual mouse sera were tested additionally to the mixed serum. Immuno-Blot analysis was performed as a second line of evidence. (b) shows IgG1, -2a, -2b, and -3 titres of mixed mouse sera in comparison for the three adjuvant substances including controls. Titres of <10^2^ have not been determined and thus are not depicted. NS = nonsignificant; *∗* = significant difference *p* < 0.05; OMVs = bacterial outer membrane vesicles; CT = cholera toxin; i.n. = intranasal vaccination; s.c. = subcutaneous vaccination.

**Figure 3:**
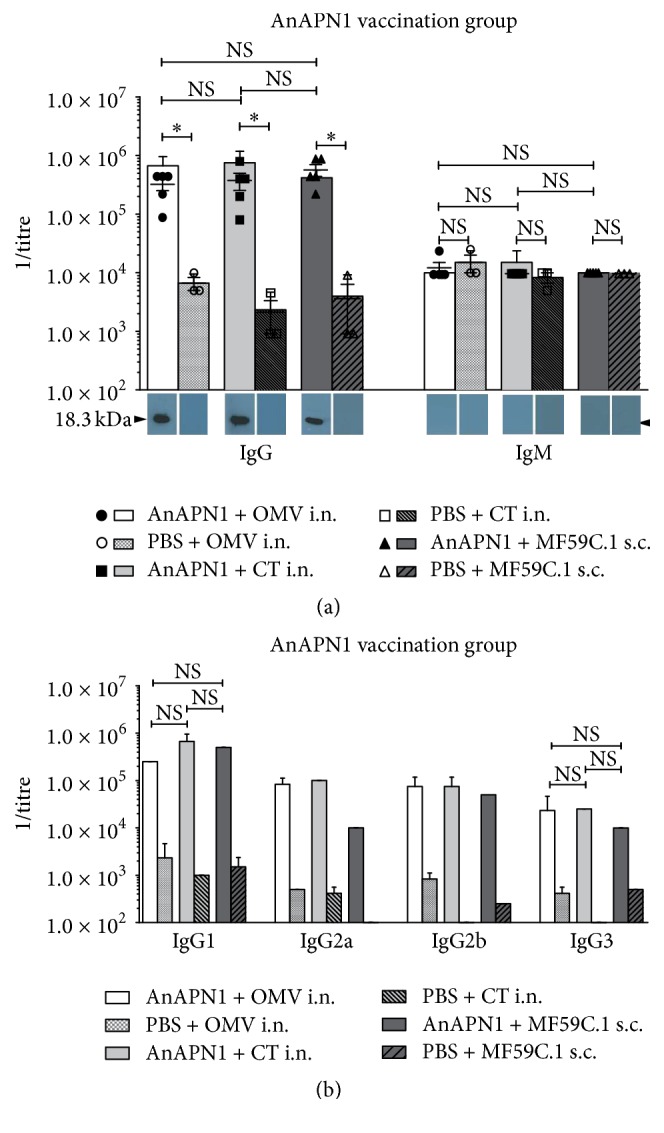
Characterization of the humoral response to AnAPN1 vaccination. Each column depicts a representative vaccination group consisting of five mice, respectively. (a) depicts whole IgG and IgM titres in direct comparison between the three adjuvants. Single mouse as well as mixed mouse serum data is shown including standard deviations. For control groups, only three individual mouse sera were tested additionally to the mixed serum. Immuno-Blot analysis was performed as a second line of evidence. (b) shows IgG1, -2a, -2b, and -3 titres of mixed mouse sera in comparison for the three adjuvant substances including controls. Titres of <10^2^ have not been determined and thus are not depicted. NS = nonsignificant; *∗* = significant difference *p* < 0.05; OMVs = bacterial outer membrane vesicles; CT = cholera toxin; i.n. = intranasal vaccination; s.c. = subcutaneous vaccination.

*(I) Humoral Immune Response.* Serum antibody titres were determined with ELISA. Titres of total IgG and IgM as well as the subclasses IgG1, IgG2a, IgG2b, and IgG3 were determined for each respective antigen and administration route. For specific serum antibody detection, Nunc Immuno Plates (Maxisorp F96 flat bottom plate, Sigma-Aldrich, Wiesbaden, Germany) were coated with the respective antigen by incubation of 100 *μ*L of antigen at 20 *μ*mol/mL in carbonate-bicarbonate buffer at 4°C overnight. The plate was subsequently washed twice with PBS containing 0.05% Tween 20 (PBS-T). Nonspecific bindings were saturated with 200 *μ*L of blocking buffer (1% bovine serum albumin in PBS with 0.05% Tween 20) for 1 h at 37°C. Serum samples were serially diluted in blocking solution, starting with a 100-fold dilution, and measured in triplicate (some individual mouse sera were measured in duplicate because of small sample volume). Endpoint dilution data from a series of 16 steps between 1 : 10^2^ and 1 : 10^6^ are presented in Figures 1–3. A 100 *μ*L of diluted serum was incubated for 1 h at 37°C, followed by four PBS-T washing steps. After addition of the secondary antibodies, plates were incubated for 1 h at 37°C and subsequently washed again four times with PBS-T. Secondary antibodies conjugated with horseradish peroxidase (HRP) were used for detection. Read-out was performed with o-phenylenediamine substrate (Sigma-Aldrich, Wiesbaden, Germany) in 0.1 M phosphate/citrate buffer (pH 5.0). The substrate was incubated for 30 min at room temperature (21 ± 1°C) under light protected conditions. Absorbance was measured with a plate reader (Tecan, Grödig, Austria) at a wavelength of 492 nm according to the instructions of the manufacturer. Secondary antibodies were chosen depending on the desired measurement. Whole IgG titres were determined using goat anti-mouse IgG (Sigma-Aldrich, Wiesbaden, Germany) diluted 1 : 1,000 in blocking solution; anti-IgM specific and anti-IgG subclass specific antibodies (goat anti-mouse IgM, IgG1, IgG2a, IgG2b, and IgG3) were purchased at Santa Cruz Biotechnology (Santa Cruz, CA, USA) and used in a dilution of 1 : 3,000 in blocking buffer.

Statistical analysis was performed with the mean optical density (OD) of each triplicate. A titre measurement was considered positive when the mean OD value was greater than the mean OD of the negative controls plus 10 standard deviations (SD). 

*(II) Cellular Immune Response.* After completion of the vaccination experiment, mice were sacrificed and spleens were aseptically removed. Spleens were lysed in lysis buffer (Passive Lysis Buffer, Promega, Madison, MI, USA) and homogenized in RPMI 1640 with 10% FCS, 1% Pen/Strep, and 1% L-Glutamine at a concentration of 7.5 × 10^6^/mL per well in 200 *μ*L. Cell count was determined with a Neubauer-chamber. Single spleen cell suspensions of the vaccinated mice were pulsed over night with the relevant proteins at the indicated concentrations (AnAPN1, 0.1 mg/mL; Pfs48/45, 0.1 mg/mL; OVA, 0.5 mg/mL; or the equivalent volume of PBS). Cells were restimulated with PMA (50 ng/mL, Sigma-Aldrich, Wiesbaden, Germany) and Ionomycin (1 *μ*g/mL, Sigma-Aldrich) for 5 h with the addition of Brefeldin A (1 *μ*g/mL, Sigma-Aldrich) after 1 h. Cells were surface stained with anti-CD3-Pe-Cy7 (clone 145-2C11, Biolegend, San Diego, CA, USA), anti-CD4-PerCP (clone GK1.5, Biolegend), and anti-CD8-APC-Cy7 (clone 53-6.7, Biolegend). Cells were fixed and permeabilised using a Foxp3/Transcription Factor Staining Buffer Set (eBioscience, San Diego, CA, USA). Intracellular staining was performed with anti-IFN-*γ*-PE (clone XMG1.2, Biolegend), anti-IL-17-FITC (clone TC11-18H10.1, Biolegend), and anti-Foxp3-Pacific Blue (clone FJK-16s, eBioscience). Cells were analysed using a BD FACS Canto II (BD Biosciences, Franklin Lakes, NJ, USA).

### 2.6. Statistical Analyses

Individual data sets were analysed using the SigmaStat Software package (Systat Software GmbH, Erkrath, Germany). ELISA data were compared with the Mann-Whitney rank sum test, as data was not always normally distributed; flow cytometric data was analysed using Student's *t*-test. *p* values of <0.05 were considered statistically significant and marked with *∗*.

## 3. Results

### 3.1. Preparation of AnAPN1, Pfs48/45, and OMV

The G/C contents of AnAPN1 and Pfs48/45 sequence after codon harmonization were 49.19% and 41.63%, respectively (see Text S1 in the Supplementary Material available online at http://dx.doi.org/10.1155/2016/3576028). Protein properties can be found in more detail in the Supplementary Material (Text S2). Codon usage was significantly improved for enterobacterial preference in the synthetic genes. Expression and purification were performed as described in more detail in Section 2 (see also Text S3). Induction in transgenic* E. coli* yielded sufficient protein amounts for vaccination experiments; the concentration of vaccine protein in the final elution sample was 4.5 mg/mL for AnAPN1 and 3.2 mg/mL for Pfs48/45, respectively. The lipopolysaccharide content of the protein preparations was determined and found to be equal to an LPS load for each administration (10 *μ*g) of 0.02 ng in the case of AnAPN1 and 0.03 ng for Pfs48/45, respectively. OMVs were produced as described above and before [29]. Emulsified OMVs were diluted to a concentration of 5 × 10^6^/*μ*L for the use as adjuvant.

### 3.2. Immunization

In each vaccination group, a total of five mice were vaccinated. Individual experiments were repeated twice independently. Vaccinations were well tolerated without any noticeable local or systemic adverse events. Laboratory animals were observed daily and with special scrutiny the hours following the administration of the vaccines. Standardized score sheets were used to document behaviour and weight. No significant difference in weight gain or behaviour could be observed between groups; no signs of acute toxicity could be observed.

### 3.3. ELISA

In the OVA groups, total IgG titres reach up to 1 : 10^6^, equally distributed between the i.n. vaccination routes (Figure 1). The titres of s.c. vaccination with MF59C.1 were significantly lower than with both i.n. routes. For all three vaccines, IgG1 is the main IgG subclass evoked, again with the intranasal routes being superior to the s.c. administration. IgG3 subclass titres were found at similar levels compared to IgG1 for all vaccine routes. IgM titres are only roughly one order of magnitude below IgG titres even after one month. All titres increased significantly during the course of vaccination. Detectable IgM persisted throughout the experiment in the groups of all three adjuvants. Although the overall IgG titres in the MF59C.1 group were lower, the IgM titres were not significantly different in all three groups.

In the groups vaccinated with Pfs48/45, total IgG titres were in the range of >10^5^-10^6^ (Figure 2).

In Western Blot analysis, a strong IgG reaction can be detected. Interestingly, a higher molecular mass band can be seen in the OMV i.n. Pfs48/45 group and to a lesser extent in the other Pfs48/45 vaccinated groups. It could not be detected in the mock-immunized groups. Mice vaccinated with i.n. OMV and to a lesser extent those vaccinated with MF59C.1 had persistent IgM and low IgG3 titres. However, substantial IgM titres were observed in the ELISA measurements also of the mock-immunized groups. Thereby, the Western Blot analysis could only detect a specific reaction in the Pfs48/45 groups immunized with OMV as well as MF59C.1, which also demonstrated a significantly higher titre in the vaccinated group as compared to the mock-immunized control (Figure 2). In contrast, mice vaccinated with i.n. CT had no persistent IgM but had higher IgG3, with titres for IgG3 being higher than those for IgG2b. Most of the IgG response was of the IgG1 subclass in all vaccination groups. OMV vaccinated mice had, however, lower IgG1 titres compared to the other groups. Overall, titres achieved with the s.c. vaccination were significantly higher than in the other two groups.

Mice vaccinated with AnAPN1 developed high titres of up to 10^6^ (Figure 3). Mucosal vaccination showed a trend towards higher titres compared to the parenteral vaccination. For all groups, no IgM persistence above the titres of the mock-immunized groups could be observed and IgG3 titres were lower than IgG2a and -b titres. MF59C.1 vaccination induced the lowest IgG2a titres.

When controlling all titres against a decoy protein (DHFR of the mouse) with identical N-terminal signal sequences and His tag as used in AnAPN1 and Pfs48/45, respectively, no deviation from background could be detected in any group, excluding unspecific binding to the purification tag sequences.

### 3.4. T Cell Response

**Figure 4:**
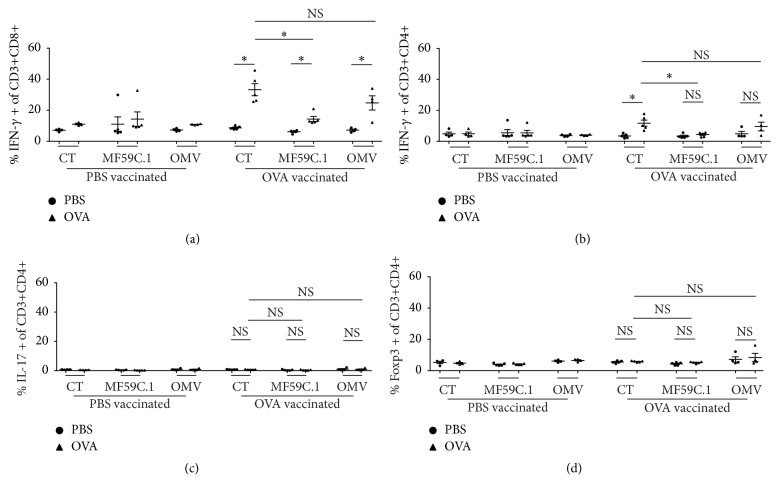
Characterization of the cellular response to ovalbumin vaccination. Each dot or triangle represents an individual mouse. Each PBS vaccination consisted of four mice and each ovalbumin vaccination group of five mice. Comparisons between groups were performed by Student's *t*-test. (a) Percentage of IFN-g secreting CD3 and CD8 double positive cells. (b) Percentage of IFN-g secreting CD3 and CD4 double positive cells. (c) Percentage of IL-17 secreting CD3 and CD4 double positive cells. (d) Percentage of Foxp3 positive CD3 and CD4 double positive cells. NS = nonsignificant; *∗* = significant difference *p* < 0.05; OMVs = bacterial outer membrane vesicles; CT = cholera toxin.

OVA was used as model antigen, in order to analyse the ability of the used adjuvants (CT, MF59C.1, and OMV) to induce an antigen-specific cellular response. All three adjuvants were able to induce OVA-specific cytotoxic T cells (Figure 4(a)). CT was better than MF59C.1 and showed a trend towards stronger induction of cytotoxic T cells than OMV (Figure 4(a)). CT was the only adjuvant able to induce a Th1 response towards OVA (Figure 4(b)), while MF59C.1 and OMV did not. None of the adjuvants induced a significant number of OVA-specific Th17 or regulatory T cells (Figures 4(c) and 4(d)).

**Figure 5:**
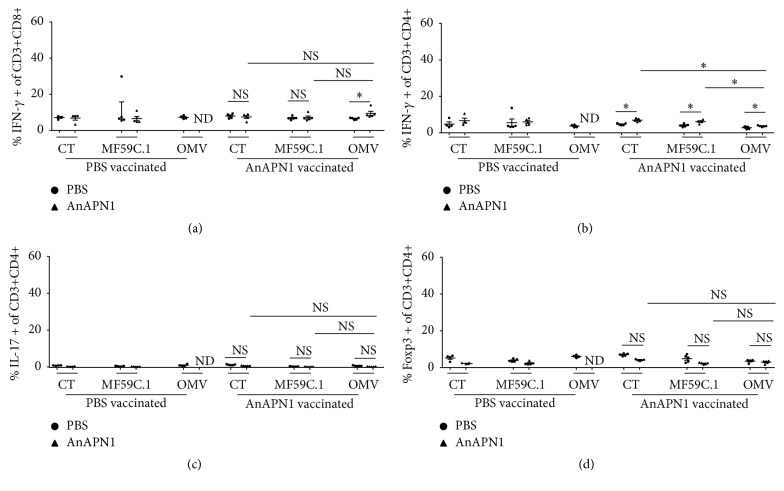
Characterization of the cellular response to AnAPN1 vaccination. Each dot or triangle represents an individual mouse. Each PBS vaccination consisted of four mice and each AnAPN1 vaccination group of five mice. Comparisons between groups were performed by Student's *t*-test. (a) Percentage of IFN-g secreting CD3 and CD8 double positive cells. (b) Percentage of IFN-g secreting CD3 and CD4 double positive cells. (c) Percentage of IL-17 secreting CD3 and CD4 double positive cells. (d) Percentage of Foxp3 positive CD3 and CD4 double positive cells. NS = nonsignificant; *∗* = significant difference *p* < 0.05; OMVs = bacterial outer membrane vesicles; CT = cholera toxin; ND = not determined.

In contrast, when comparing the ability of the said adjuvants to induce AnAPN1-specific cellular responses, only OMV was able to induce a cytotoxic T cell response towards AnAPN1, but all induced a Th1 response towards the antigen (Figures 5(a) and 5(b)). Again, no induction of antigen-specific Th17 or regulatory T cells (Figures 5(c) and 5(d)) could be observed.

When assessing the responses induced by Pfs48/45 vaccination, we found a decrease in cell number for all the conditions where cells have been restimulated with Pfs48/45 (Figure S4). All cell populations including cytotoxic T cells, Th1 cells, Th17 cells, and regulatory T cells decreased upon restimulation with Pfs48/45 (Figure S4).

## 4. Discussion

In the present study, humoral and cellular immune responses evoked by different vaccination methods using malaria transmission blocking antigens and ovalbumin in mice have been characterized and compared [15–27]. This study is the first to compare humoral and cellular immune responses for i.n. OMV adjuvating malaria transmission blocking antigens. Purified antigens from transgenic bacterial cultures (or purchased, in the case of OVA) were used and mixed with the respective adjuvant. This strategy does not make use of the full capacity of OMV for vaccination, as the antigens can be expressed directly inside the OMV [31]. To allow direct comparison of the vaccination routes, the same antigen batch was used with the different adjuvants. This approach considerably reduces possible artefacts due to folding abnormalities or contaminations of the used antigen preparation. However, this cannot control for antigen losses, which are to be expected due to uptake on the mucosal surface or sneezing out of the vaccination solution by the animals. To allow for a better evaluation of our results and comparison with literature, three previously studied antigens have been used. In this study, an adjuvant licensed for human use (MF59C.1) [32] and two mucosal adjuvants were used. While cholera toxin cannot be used in humans due to toxicity, it is considered highly potent and can be used as the reference substance in mice [33].

Previously, the use of modified OMV for i.n. vaccination has been reported and also been used as intranasal adjuvant [12], but concerns about their endotoxin (LPS) content is seen as problematic especially for parenteral application. To circumvent this issue, injectable formulations use extracted OMV without large amounts of LPS [34]. Mucosal surfaces may be less sensitive to LPS, since these are generally colonized by bacteria and thus potentially more amenable to safe OMV usage [35].

Humoral immune responses are key for the success of classic vaccine antigens [3]. For the malaria transmission blocking antigens AnAPN1 and Pfs48/45, antibodies are pivotal for transmission blockade in the midgut of the mosquitoes and IgGs seem to be of primary importance [15–27]. Although, for malaria transmission blocking vaccines, the long-term antibody response is important and has not been studied here, antibodies evoked at the peak of the immune response are of interest, offer valuable first insights into immunogenicity, and help with the design of studies covering the more antigen-specific application areas.

In the OVA vaccination groups, the total IgG titres of the i.n. groups were significantly higher than in the MF59C.1 adjuvated, s.c. administered animal batch (Figure 1). Injected OVA was previously found to be very immunogenic [36]. These high titres after i.n. vaccination are surprising, as OVA administered intranasally with liposomes was previously found to be less efficient than in combination with CT [37, 38]. High IgG3 titres, which were significantly lower than IgG1 in all groups, and persistent IgM were observed as well (Figure 1). This is noteworthy as two of the protocols involved the i.n. application of the antigen/adjuvants mixture rather than the injection. Therefore, it is possible that the properties of OVA as an antigen influenced the antibody subclass distribution rather than the application route or adjuvant substance. Other studies using different adjuvant substances reported IgG2a titres to be higher than IgG3 when injected with CpG, Bp, and CFA, respectively [36]. In these studies, however, i.n. routes have not been used at all and a clear influence of the adjuvant substance can be seen. OVA groups show a strong Th2 dependent response with IgG1 as the most dominant subclass (Figure 1). Nevertheless, IgG2a titres are only one order of magnitude lower in titre levels than IgG1 and are still in the range of 1 : 10^5^ (Figure 1). This argues for a strong Th1 answer as well [39]. Besides these two, the T cell independent IgG3 reaches almost the same level as IgG1, which is noteworthy.

Titres in the Pfs48/45 groups were on average similar to the OVA titres, however, with a tendency to be lower especially in the i.n. groups (Figure 2). The s.c. group vaccinated with MF59C.1 actually showed the highest titre measured in the experimental series described here. The titres for total IgG were not different between the i.n. groups, however, significantly higher in the s.c. group (Figure 2). In Western Blot analysis, a higher molecular weight band can be appreciated in the OMV i.n. Pfs48/45 vaccinated group and to much less extent also in the MF59C.1 and CT groups (see Figure 2(a)). This band is most likely a very slight reaction against traces of larger molecular weight protein found within the purified Pfs48/45 stock (see also silver stain in Supplementary Material (S3)), which is most prominently seen in the OMV vaccinated group. However, it is not present in the controls; thus a significant unspecific reaction can be excluded. Interestingly, IgM titres persisted significantly above the controls in the OMV i.n. group and the MF59C.1 group but not in the CT group (Figure 2). The OMV adjuvated i.n. administration also has low IgG3 levels as well as relatively low IgG2a levels (Figure 2). Therefore, the antibody response in this route seems to be mainly Th2 based. In contrast, the MF59C.1 groups showed significantly higher IgG3 titres (Figure 2). Thus, besides the strong Th2 mediated IgG1 response T cell independent IgG3 seems to be of greater importance than with the OMV adjuvated i.n. group. The CT i.n. group shows an intermediate phenotype as compared to the two other groups. Interestingly, the OMV adjuvant was able to stimulate a strong IgG3 response in the OVA-experiment and also strong Th1 mediated IgG2a titres in the OVA as well as AnAPN1 group (see Figures 1 and 3, resp.). IgG3 has been described before to be the second most important antibody subclass evoked after vaccination with* Neisseria* OMV [40]. The lack of IgG3 and IgG2a has to be attributed not to the properties of the adjuvant itself but rather to the combination of the adjuvant with the antigen, or possible penetration, or uptake problems of the antigen Pfs48/45 when administered in the i.n. application route. The overall size of the Pfs48/45 protein construct was relatively large with 49.4 kDa and charge is comparatively strong (−10.8 at pH 7), whereas AnAPN1 is much smaller and with less charge (18.3 kDa and −1.2 at pH 7), and OVA, although of roughly the same size (45 kDa), is favourable regarding solubility and charge (Text S2). Still, the humoral immune response was strong in all groups, never reaching titres below 1 : 10^5^ (Figures 1–3).

Overall the OVA and Pfs45/48 vaccinations had a tendency of reaching higher average titre levels over all three vaccination routes (both 1.1 × 10^6^) as compared to AnAPN1 (6.1 × 10^5^) (Figure 3), arguing for all three application routes to be a relatively robust system, although the difference in size and physical properties are striking (Text S2). The AnAPN1 vaccination groups do not show significant differences between the application routes regarding the total IgG titres (Figure 3). There seems to be a trend towards higher titres on the side of the i.n. groups as compared to the s.c. group, but this was not significant. All groups lean towards an IgG1 based response, which is most dominant in the MF59C.1 group (Figure 3). Other than with OVA, the different subclass distribution patterns argue for a more Th1/2 balanced response with the OMV group and a mostly Th2 biased answer in the MF59C.1 group. All three groups show only relatively low IgG3 levels; thus T cell independent IgG3 might not be easily generated with AnAPN1, which is in accordance with a previous study using incomplete Freund's adjuvant and Alhydrogel in BALB/c and Swiss Webster mice [17]. This may be due to properties of the antigen itself rather than the adjuvant substance, as high IgG3 levels could be detected with all three adjuvants when applied together with OVA. Subclass antibody data published previously for Pfs48/45 vaccination trials also showed dominating IgG1 responses with highly variable contributions of IgG2a, IgG2b, and IgG3 depending on adjuvants (Montanide ISA-51, Alum, CFA) and/or animal models (olive baboons, BALB/c mice) used [26].

The induction of T cell responses through vaccination does not only rely on the adjuvant and the administration route used, but also rely on the target antigen and its amenability to MHC-I or II presentation [41, 42]. All adjuvants of the present study were able to support cytotoxic T cell responses towards OVA (Figure 4(a)). However, only OMVs could induce such T cells against AnAPN1 (Figure 5(a)). Therefore, the difference observed with this antigen cannot be explained by the general capacity of the adjuvant to induce CD8 T cell responses, but rather by the nature of the antigen which may not be readily accessible to the MHC presenting machinery and requires adequate maturation and stimulation of antigen-presenting cells for proper immune induction as was described for other antigens [43]. A lack of proper presentation of AnAPN1 during restimulation* in vitro* can be ruled out, since reactive T cells could be detected in the case of OMV. In this setting, OMVs seem to mediate best antigen presentation of AnAPN1* in vivo* leading to measurable immune responses compared to the other adjuvants. It is also important to note that no induction of antigen-specific regulatory T cells could be found which would have hampered the function of effector T cells and thus hijacked any therapeutic benefit [44]. CD8 positive T cells may be important for malaria protection but are not needed for malaria transmission blocking activity [15–27, 45]. However, levels can be used to better characterize the immune response supported by the individual route. The data, however, cannot preclude an advantage over other types of vaccination based on the induction of CD8 T cell responses [46].

While IgG1 was the most prominent antibody subtype detected for all antigens and adjuvants, only Th1 but not Th2 cellular responses could be detected upon restimulation. Th2 cells may be below the limit of detection. The analysis of antibody subtypes at the time point of sampling also revealed persistence of IgM, potentially indicating an incomplete class switch towards IgG at that time point. The analysis is, however, somewhat hampered by the relatively high background titres observed in the mock-immunized adjuvant control groups which may demonstrate increased unspecific reaction in the directly coated ELISA. The increased titres in the mock controls were observed mainly with the Pfs48/45 and AnAPN1 groups and to much less extent in the OVA group (Figures 1, 2, and 3). The Western Blot controls performed in parallel only displayed a specific but weak reaction in the Pfs48/45 groups immunized in conjunction with OMV or MF59C.1 (Figure 2). Such delayed isotype switch has been described upon infection [47]. Cellular responses against Pfs48/45 could not be detected due to toxicity of the protein when used in the restimulation protocol (Figure S4). Pfs48/45 cytotoxicity* in vivo*, however, seems not to be relevant. The detected antibody response requires CD4 T helper cell support and thus proves the existence and function of these T cells in vaccinated mice [48]. Furthermore, no significant side effects or reduced weight gain was observed in the experimental groups confronted with Pfs48/45 compared to the other antigens and the application controls arguing for negligible toxic effects on the mouse* in vivo*.

## 5. Conclusions

The results of this study argue that, based on the antigen-adjuvant cocktail chosen, humoral and cellular immune responses may be shaped differently. They also suggest that i.n. vaccination with OMV may be a potent strategy to enhance intranasal heterologous vaccine antigens. OMV-evoked antibody titres and T cell responses were about as strong as after i.n. immunization with CT. This aspect is of special importance as OMV application to the nasal mucosa may eventually be considered an easy and safe vaccination route as compared to CT, which is not used in humans due to toxicity. Importantly, i.n. vaccination led to robust and reproducible immune responses, indicating that antigen application and uptake do not limit the approach, even using antigens of large size and with strong charge such as Pfs48/45. Parenteral applications such as the MF59C.1 adjuvated group used here provide an almost complete antigen bioavailability; however, the immune responses were not found to be significantly better than with mucosal routes with uncertain bioavailability. The results provide the rationale for further improvement and engineering of OMV to facilitate their adherence to mucosal surfaces and boost of the immune response. Further studies are also aiming at directly coupling (malaria) antigens to OMV and at generating transgenic bacterial strains where supernatant can be used directly as a vaccine for use in resource limited settings.

## Supplementary Material

In the Supplementary Material, more information on the optimized sequences (Text S1), the protein properties (Text S2) and the expression as well as purification processes (Text S3) of AnAPN1 and Pfs48/45 can be found. Additionally, the cellular immune response to Pfs48/45 vaccination is depicted in Figure S4.
