# Supplementary material for: Comparison of Intranasal Outer Membrane Vesicles with Cholera Toxin and Injected MF59C.1 as Adjuvants for Malaria Transmission Blocking Antigens AnAPN1 and Pfs48/45
Source: J Immunol Res. 2016 Apr 27;2016:3576028. doi: 10.1155/2016/3576028 (PMC4863099; doi:10.1155/2016/3576028)
Supplement: Supplementary file 1 — In the Supplementary Material, more information on the optimized sequences (Text S1), the protein properties (Text S2) and the expression as well as purification processes (Text S3) of AnAPN1 and Pfs48/45 can be found. Additionally, the cellular immune response to Pfs48/45 vaccination is depicted in Figure S4. [file 3576028.f1.pdf]

Text S1:

A: AnAPN1:

Optimized Sequence: **% C+G = 49.19**

GGATCCGGTACCCTCGAGACAGAACGTTATCGTCTGCCGACCACCAGTATTCCGA
TTCATTATGATCTGCATCTGCGCACCGAAATTCATCGTAATGAACGTACCTTTACC
GGCACCGTTGGTATTCAGCTGCAAGTTGTTCAGGCAACCGATAAACTGGTGATGC
ATAATCGTGGTCTGGTTATGAGCAGCGCAAAAGTTAGCAGCCTGCCGAATGGTGT
TACAGGTGCACCGACCCTGATTGGTGATGTTCAGTATAGCACCGATACCACCTTT
GAACATATTACCTTTACAAGCCCGACCATTCTGCAACCGGGTACATATCTGCTGG
AAGTTGCATTTCAGGGTCGTCTGGCAACCAATGATGATGGTTTTTATGTTAGCAG
CTATGTGGCCGATAATGGTGAACGTCGTTATCTGTAAGATATCCTGCAGAAGCTT

B: Pfs45/48:

Optimized Sequence: **% C+G = 41.63**

GGATCCGGTACCCTCGAGAATAATGATTTCTATAATCCGAGCGCACTGAACAGCG
AAATTAGCGGTTTTATTGGCTACAAATGCAACTTTAGCAATGAAGGCGTGCATAA
TCTGAAACCGGATATGCGTGAACGTCGTAGCATTTTTTGTAATATCCACAGCTAC
TTCATCTATGATAAAATCCGCCTGATCATCCCGAAAAAAAGCAGCAGCCCTGAGT
TTAAAATCCTGCCGGAAAAATGCTTCCAGAAAGTGTATACCGATTATGAAAATCG
CGTGGAAACCGATATTAGCGAACTGGGTCTGATTGAATATGAGATCGAAGAAAA
TGATACCAACCCGAACTATAACGAACGCACCATTACCATTAGCCCGTTTAGCCCG
AAAGATATTGAATTTTTCTGCTTCTGCGACAACACCGAAAAAGTGATTAGCAGCA
TTGAAGGTCGTAGCGCAATGGTTCATGTTCGTGTTCTGAAATATCCGCACAACAT
TCTGTTTACCAATCTGACCAATGACCTGTTTACATATCTGCCGAAAACCTATAACG
AGAGCAATTTTGTTAGCAACGTTCTGGAAGTGGAACTGAATGATGGTGAACTGTT
TGTTCTGGCATGTGAGCTGATCAACAAAAAATGTTTTCAAGAAGGCAAAGAAAA
AGCCCTGTATAAAAGCAACAAAATCATCTATCATAACAAACTGACCATCTTTAAA
GCACCGTTTTACGTGACCAGCAAAGATGTTAATACCGAGTGTACCTGCAAATTCA
AAAACAACAACTATAAAATCGTGCTGAAACCGAAATATGAAAAAAAAGTTATCC
ACGGCTGCAACTTCAGCAGCAATGTTAGCAGCAAACATACCTTTACCGATAGCCT
GGATATTAGCCTGGTTGATGATAGCGCACATATTAGCTGTAATGTTCATCTGAGC
GAGCCGAAATATAACCATCTGGTGGGTCTGAATTGTCCGGGTGATATTATCCCGG
ATTGTTTTTTTCAGGTTTATCAGCCGGAAAGCGAAGAACTGGAACCGAGCAATAT
TGTTTATCTGGATAGCCAGATTAATATCGGCGACATCGAGTATTATGAAGATGCC
GAAGGTGACGACAAAATCAAACTGTTTGGTATTGTTGGCAGCATTCCGAAAACCA
CCAGTTTTACCTGTATCTGCAAAAAAGACAAAAAAAGCGCCTATATGACCGTGAC
CATTGATAGCTGAGATATCCTGCAGAAGCTT

Text S2:

A: AnAPN1:

Molecular Weight 18331.50 Daltons; 163 Amino Acids; 15 Strongly Basic (+) Amino Acids (K, R); 18 Strongly Acidic (-) Amino Acids (D,E); 47 Hydrophobic Amino Acids (A, I, L, F, W, V); 48 Polar Amino Acids (N, C, Q, S, T, Y); 6.726 Isoelectric Point ; -1.239 Charge at pH 7.0.

MASRGSHHHHHHIEGRRDRGPEFELGTLETERYRLPTTSIPIHYDLHLRTEIHRNERTF
TGTVGIQLQVVQATDKLVMHNRGLVMSSAKVSSLPNGVTGAPTLIGDVQYSTDTTF
EHITFTSPTILQPGTYLLEVAFQGRLATNDDGFYVSSYVADNGERRYL

B: Pfs48/45:

Molecular Weight 49425.90 Daltons; 429 Amino Acids; 49 Strongly Basic (+) Amino Acids (K, R); 62 Strongly Acidic (-) Amino Acids (D, E); 125 Hydrophobic Amino Acids (A, I, L, F, W, V); 136 Polar Amino Acids (N, C, Q, S, T, Y); 5.874 Isoelectric Point ; -10.835 Charge at PH 7.0.

MASRGSHHHHHHIEGRRDRGPEFELGTLENNDFYNPSALNSEISGFIGYKCNFSNEGV
HNLKPDMRERRSIFCNIHSYFIYDKIRLIIPKKSSSPEFKILPEKCFQKVYTDYENRVET
DISELGLIEYEIEENDTNPNYNERTITISPFSPKDIEFFCFCDNTEKVISSIEGRSAMVHVR
VLKYPHNILFTNLTNDLFTYLPKTYNESNFVSNVLEVELNDGELFVLACELINKKCFQ
EGKEKALYKSNKIIYHNKLTIFKAPFYVTSKDVNTECTCKFKNNNYKIVLKPKYEKK
VIHGCNFSSNVSSKHTFTDSLDISLVDDSAHISCNVHLSEPKYNHLVGLNCPGDIIPDC
FFQVYQPESEELEPSNIVYLDSQINIGDIEYYEDAEGDDKIKLFGIVGSIPKTTSFTCICK
KDKKSAYMTVTIDS

Text S3:

AnAPN1 induction with pASK-iba37+

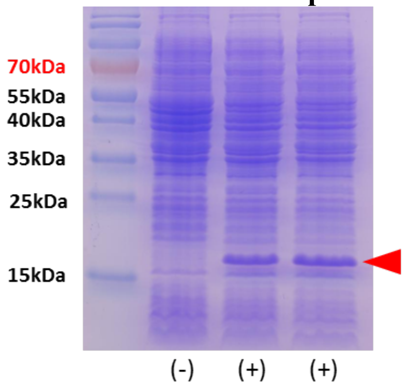

Before (-) and after (+) induction with AHT as described in materials and methods section

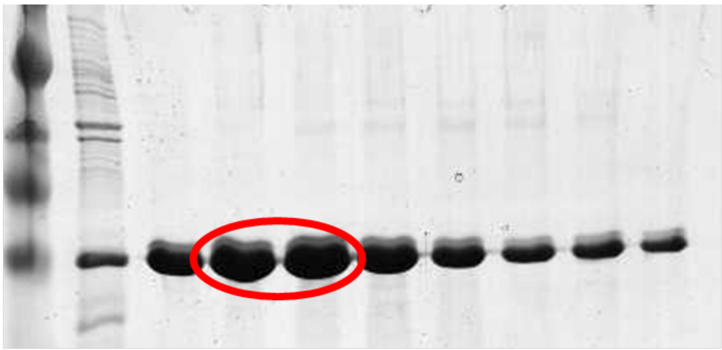

chosen purification conditions AnAPN1 from induced pellet using NiNTA column.

Pfs48/45 induction with pASK-iba37+

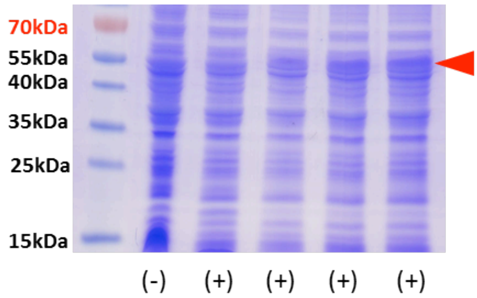

Before (-) and after (+) induction with AHT as described in materials and methods section

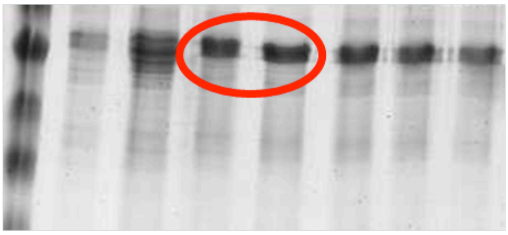

chosen purification conditions Pfs48/45 from induced pellet using NiNTA column.

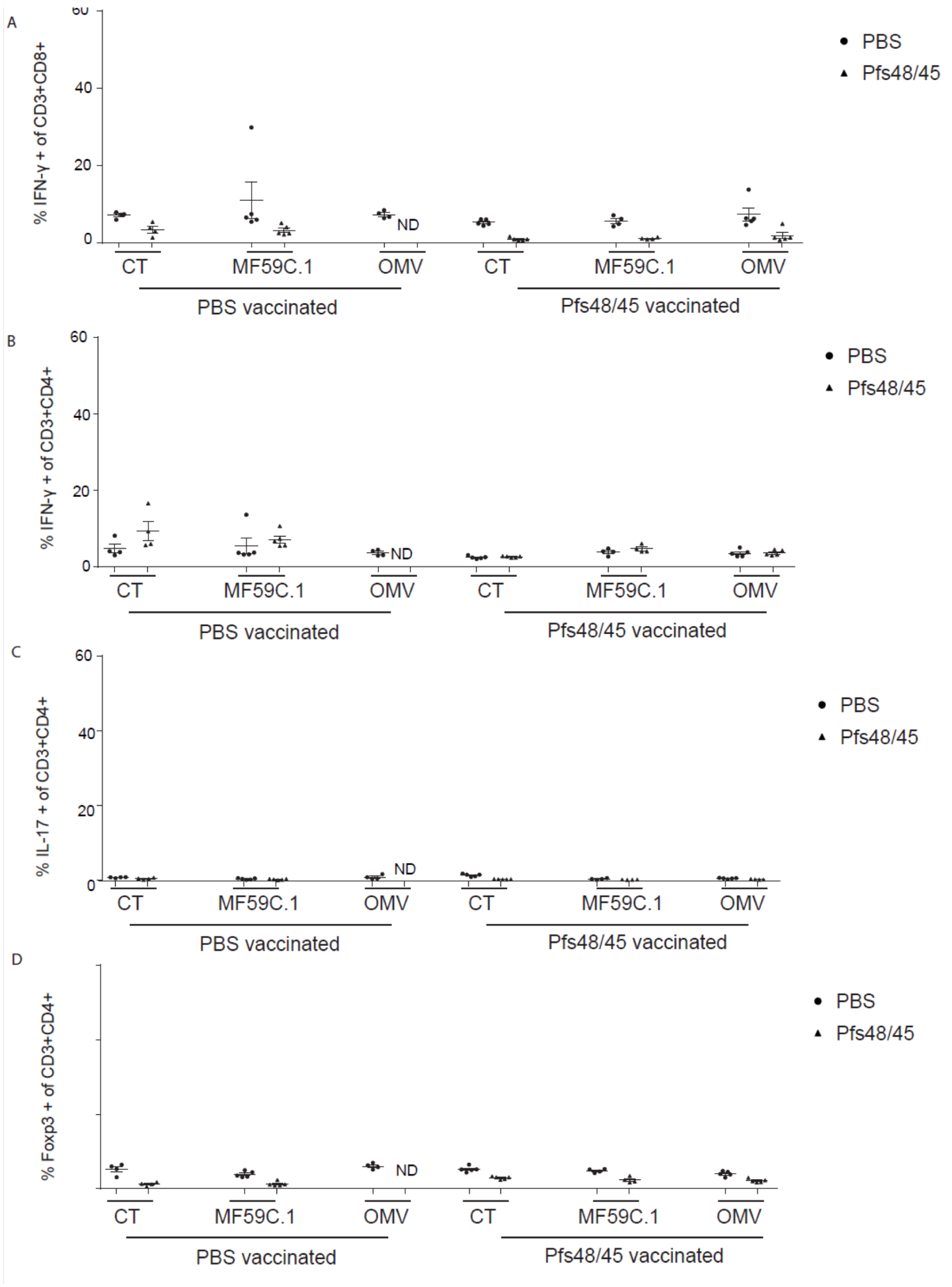

Figure S4. Characterization of the cellular response to Pfs48/45 vaccination. Each dot or triangle represents an individual mouse. Each PBS vaccination consisted of four mice and each Pfs48/45 vaccination group of five mice. (A) Percentage of IFN-γ secreting CD3 and CD8 double positive cells. (B) percentage of IFN-γ secreting CD3 and CD4 double positive cells. (C) Percentage of IL-17 secreting CD3 and CD4 double positive cells. (D) Percentage of Foxp3 positive CD3 and CD4 double positive cells. Abbreviations: OMV = bacterial outer membrane vesicles; CT = cholera toxin, ND = not determined.
